# Therapeutic Approaches in the Stimulation of the Coronary Collateral Circulation

**DOI:** 10.2174/1573403X113099990027

**Published:** 2014-02

**Authors:** Achim Degen, Dominic Millenaar, Stephan H. Schirmer

**Affiliations:** Klinik für Innere Medizin III (Kardiologie, Angiologie und Internistische Intensivmedizin), Universitätsklinikum des Saarlandes, 66421 Homburg/Saar, Germany

**Keywords:** Coronary artery disease, arteriogenesis.

## Abstract

Arteriogenesis as a way to restore blood flow after arterial occlusion has been under investigation for the treatment of coronary artery disease (CAD) for decades. Therapeutic approaches so far have included delivery of cytokines and growth factors as well as mechanical stimulation such as external counterpulsation. As knowledge on the mechanisms of arteriogenesis expanded, new therapeutic approaches have emerged.

This review summarizes recent attempts to stimulate the growth of the coronary vasculature and discusses their potential in clinical application. This article also delivers an overview of current studies and trials on coronary arteriogenesis.

## INTRODUCTION

For decades ischemic heart disease (IHD) has been the leading cause of death in industrialized western societies [1]. Macrovascular atherosclerosis leading to coronary artery obstruction and impairment of blood flow towards myocardial tissue is the underlying pathophysiology leading to IHD. Interventional and surgical revascularization strategies provide the hallmark of treatment options in this setting. Having unequivocally demonstrated improved survival in acute coronary syndromes (ACS) [2, 3], revascularization by percutaneous coronary intervention (PCI) has hitherto failed to yield prognostic benefit in stable coronary artery disease (CAD) [4]. Next to emergency revascularization, the presence of natural pre-existing bypasses improves survival in ACS [5]. Sufficiently developed collateral arteries, which are present in about one third of patients with CAD and also in about one fifth of patients without CAD [6], are associated with reduced long-term cardiac and overall mortality [7]. Therefore, stimulation of collateral artery growth (arteriogenesis) has been proposed in the management of IHD. Notably, there are several different mechanisms of vessel growth serving different purposes. While vasculogenesis describes the formation of blood vessels in embryogenesis, the term angiogenesis is used for ischemia-induced sprouting of new capillaries from existing blood vessels [8]. Finally, the term arteriogenesis has been coined to describe an increase in diameter of preexisting collateral anastomoses and their maturation into leading arterial vessels [9]. Potentially allowing bulk blood flow, only arteriogenesis is capable of supplying sufficient blood flow after occlusion of the native vessel by increasing blood flow up to factor 20 following a two-fold enlargement of a collateral artery [10, 11].

The following review describes different approaches including inflammatory as well as mechanical stimuli but also highlighting new experimental approaches and possible downsides of an increased coronary collateral vasculature.

## INFLAMMATORY CYTOKINE THERAPY – ALREADY OUTDATED?

Approaches to promote collateral artery growth have advanced from animal studies to clinical investigations studies. Arteriogenesis being an inflammatory process [12] it seemed logical to promote inflammation by systemic application of proinflammatory cytokines. First small studies based on positive data from experimental studies showed promising results. However, most randomized studies were unable to demonstrate therapeutic benefit of putative pro-arteriogenic substances when compared to placebo or showed severe side effects such as promotion of atherosclerosis and development of ACS, which is to be demonstrated below. 

Most of the studies first concentrated on angiogenesis as the main mechanism of blood flow restoration. The VIVA trial [13] (**V**ascular endothelial growth factor in **I**schemia for **V**ascular **A**ngiogenesis), in which vascular endothelial growth factor (VEGF) was tested versus placebo in IHD had neutral results. Similarly, the AGENT [14] (**A**ngiogenic **GEN**e **T**herapy) and FIRST [15] (**F**GF **I**nitiating **R**eva**S**cularization **T**rial) studies enquiring the effects of fibroblast growth factor (FGF) on capillary growth showed no benefit over placebo treatment. In the AGENT trial, an adenoviral vector containing a human fibroblast growth factor gene was applied via an intracoronary route in patients with stable IHD. Treadmill exercise testing was used to evaluate efficacy of treatment. Overall, there was a non-significant increase in exercise time between treatment and placebo group. A significant difference in the increase of exercise time was only reported in patients with baseline exercise time of less than 10 minutes. Similarly, the FIRST trial assessed single-shot intracoronary application of recombinant FGF2 (rhFGF2) for improvement of exercise tolerance in patients with coronary artery disease. Again, there was no significant difference reported in comparison to placebo treatment. The VIVA trial focused on recombinant human vascular endothelial growth factor (rhVEGF) in angina patients. Study endpoint was angina frequency and exercise time evaluated by treadmill test. The intracoronary application of rhVEGF resulted in no significant difference of angina frequency and exercise time when compared to placebo. 

In an attempt to stimulate specifically arteriogenesis which in the meantime had proven superior to angiogenesis in restoring blood flow after arterial occlusion [10, 11], granulocyte-monocyte-colony stimulating factor (GM-CSF) was administered in a small study by Seiler *et al*. [16]. GM-CSF is a colony stimulating factor mobilizing progenitor cells of the granulocyte and macrophage cell line from the bone marrow and protecting monocytes – which play an important role in arteriogenesis [17] – from apoptosis. The application of this substance had been validated in several different animal models and also showed an increase of the coronary flow index in the 11 CAD patients included in the study when compared to placebo treatment [18].

However, in a further study investigating the effects of a subcutaneous-only therapy with GM-CSF concerns about the safety of the drug arouse, with two of seven patients in the treatment group suffering an acute coronary syndrome [19]. To reduce side effects, the authors of the GM-CSF studies started investigating the effect of granulocyte-colony stimulating factor (G-CSF) only. A short-term treatment period with subcutaneous G-CSF significantly enhanced coronary collateralization as invasively measured by collateral flow index [20]. A study using pegylated G-CSF with reduced injection frequency and reduced side-effects is currently being carried out (clinicaltrials.gov identifier NCT00886509). 

Atherogenesis itself being an inflammatory process [21], attempted stimulation of arteriogenesis through induction of systemic inflammation via application of proinflammatory cytokines poses several possible risks. This notion was exemplified by experimental investigations on monocyte chemoattractant protein 1 (MCP1). While administration of MCP1 was demonstrated to increase collateral artery formation in mice, it was also shown to lead to plaque progression in dyslipidemia mouse models [22, 23]. Likewise, VEGF has been reported to enhance not only angiogenesis but also plaque progression [24].

Of the aforementioned clinical studies, one involving the subcutaneous application of GM-CSF had to be terminated prematurely when two patients developed an acute coronary syndrome [25]. Also, most of the cytokines thought to promote collateral artery growth are found at relatively high local concentrations in patients with insufficient coronary collateralization and might thus not be the ideal therapeutic target [26].

In an attempt to stimulate arteriogenesis through administration of a mediator without proinflammatory properties and the concurrent atherogenic side effects, Neuropeptide Y (NPY) was tested in swine suffering from metabolic syndrome. Treatment with NPY resulted in up-regulation of pro-arteriogenic proteins while down-regulating anti-angiogenic factors [27] and could therefore be another possible target for the stimulation of collateral artery growth in the future. However, the effects described might not specifically stimulate arteriogenesis, but also angiogenesis.

Studies involving a visual angiographic grading of collateralization and analyzing serum concentrations of inflammatory cytokines proved modulation of inflammatory cytokines in patients with good collateralization. Angiostatic ligands such as C-X-C motif chemokine 10 (CXCL10) and C-X-C motif chemokine 11 (CXCL11) were decreased, while higher concentrations of angiogenic ligands were associated with a well-developed collateral circulation [28].

## MECHANICAL STIMULATION – THE IDEAL APPROACH?

In accordance with the pathophysiology of arteriogenesis corroborating animal experiments, physical exercise was shown to have positive effects on collateral artery growth in the clinical setting [29, 30]. The additional mechanical stress on the vessel walls induced by physical exercise is believed to be one of the main mechanisms in this model. An additional inflammatory-modulating effect of exercise as the underlying mechanism, independent of shear stress, is currently under investigation (ClinicalTrials.gov Identifier: NCT01432639). Arteriogenesis unlike angiogenesis is among other factors dependent on mechanical stress, in the coronary circulation foremost fluid shear stress during diastole [31, 32]. Fluid shear stress in pre-existent collateral anastomoses is substantially increased after vascular occlusion, as in coronary artery disease. Since coronary arteries are perfused passively during diastole, there is no discernable pulse wave. Hence, circumferential wall stress, which is another mechanical factor inducing arteriogenesis [33], appears to be negligible in coronary circulation. Mechanic factors such as fluid shear stress and circumferential wall stress activate the mechanosensitive channels of the **transient receptor potential cation channel family (TrpV4) *[34]*** in the vessel walls which in turn induce inflammatory mediators [35] and concurrent attraction of mononuclear cells (MNCs) [36]. Also, enzymes such as endothelial nitric oxide synthase (eNOS) and MNC-based inducible nitric oxide synthase (iNOS) are activated, leading to a proarteriogenic milieu [37]. Induced factors include MCP1, transforming growth factor-beta (TGFß) and adhesion molecules such as intercellular adhesion molecule (ICAM) and vascular cell adhesion molecule (VCAM). These lead to further infiltration with circulating monocytes, which initiate extracellular matrix degradation [38]. Simultaneously, liberated nitric oxide (NO) triggers expression of Actin-binding rho activating protein (ABRA) [39] and thus induces a proliferative phenotype in vascular smooth muscle cells [9]. Consequently, collateral artery growth occurs, leading to typical corkscrew-shaped conducting arterioles [36]. Generally, this growth is associated with a process called pruning, leading to obliteration of smaller collaterals and selection of fewer, but larger arteriolar vessels [40]. However, sufficient physical exercise inducing collateral artery growth seems difficult for patients with recurring and severe angina or patients suffering from heart failure.

**Fig. (1):**
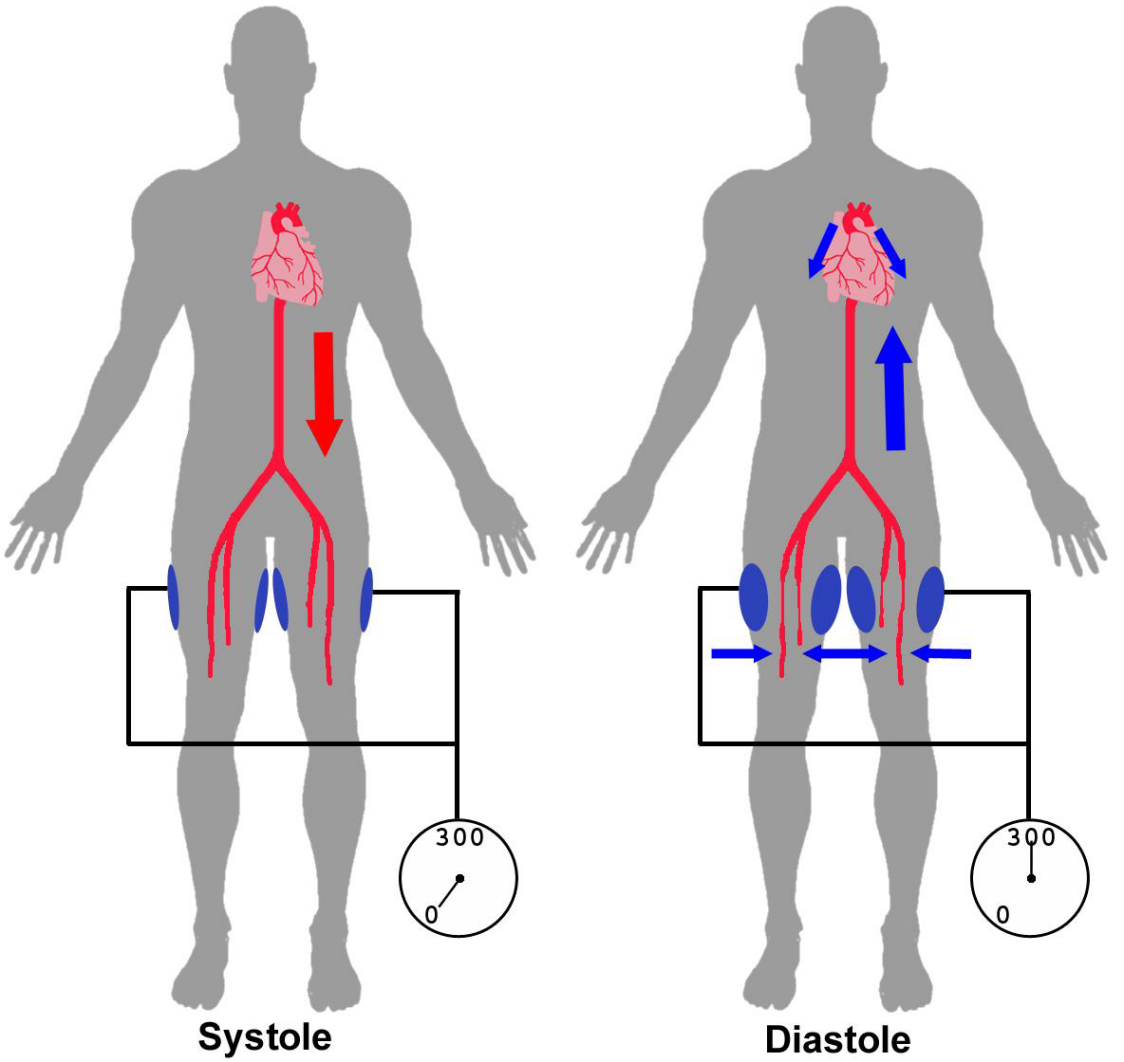
Principle of ECP. During systole pressure cuffs, which are applied to the complete lower limb, are deflated allowing normal arterial blood flow in the lower limbs. During diastole cuffs are inflated to 300mmHg causing retrograde aortic blood flow and increased myocardial perfusion, consequently leading to an increase in coronary vascular shear stress.

Given the fact that mechanical factors influence arteriogenesis [33], there has been increasing focus on a treatment that has been primarily used in the management of refractory angina [41]. Because of the difficulties of active exercise training, a passive method of blood flow augmentation has been investigated. During enhanced external counterpulsation (ECP), external hydraulic pressure of up to 300 mmHg is applied to the lower limbs at three different places triggered by electrocardiogram (ECG), causing a diastolic augmentation of coronary blood flow and increasing cardiac output, by raising preload (venous return) and lowering afterload (diastolic augmentation of aortic perfusion pressure) (Fig. **1**). In the MUST-EECP-study (**MU**lticenter **ST**udy of **E**nhanced **E**xternal **C**ounter**P**ulsation) an increase of coronary flow reserve and alleviation of angina were reported after treatment with ECP [42]. This is possibly due to the increased diastolic blood flow in the main coronary artery through increased cardiac preload and thus enhanced passive myocardial perfusion but also due to enhanced collateral artery growth based on increased fluid shear stress in pre-existing collateral anastomoses.

Two studies have directly tested the hypothesis that ECP enhances coronary collateral artery growth. 

In the non-randomized Art.Net.-2 (**Art**eriogenesis **Net**work) trial patients with stable coronary artery disease underwent a 35 1-h sessions of ECP in 7 weeks [43]. They showed an increased coronary flow index (CFI) and fractional flow reserve (FFR) as well as an improvement in clinical endpoints such as angina frequency and dyspnea after ECP.

Another investigation by Gloekler *et al*. on patients with chronic stable CAD contained a sham group (80 mmHg; mean diastolic pressure was 73 mmHg at baseline) next to an ECP-group (300 mmHg) [44]. Patients were exposed to 30 h of ECP spread over 4 weeks (twenty 90 min sessions). Since 80 mmHg cuff pressure applied in the sham group was only negligibly higher than diastolic pressure, and therefore has only little effect, it seems that venous return has the least impact, compared to diastolic augmentation. This pressure would still increase venous return to the right atrium, but could not interfere with the arterial system, being well below diastolic blood pressure. Primary endpoint was the collateral flow index (CFI) which increased in ECP-treated patients compared to sham: CFI changed from 0.125 at baseline to 0.174 at follow-up in the ECP group, while it did not improve significantly in the sham group.

In an attempt to reduce angina in symptomatic CAD-patients not suitable for revascularization, the Coronary Sinus Reducer was implanted in fifteen patients. The Coronary Sinus Reducer is an expandable stainless steel stent inserted percutaneously through the internal jugular vein narrowing the coronary sinus (CS) and thus increasing coronary venous pressure. After 6 months, angina was reduced as well as myocardial ischemia as evaluated by dobutamine echocardiography and thallium single-photon emission computed tomography (SPECT) [45]. Considering the data from this study, implantation of a Coronary Sinus Reducer could be a possible treatment not only for the alleviation of symptoms in angina patients, but also in the improvement of myocardial perfusion.

An active means of increasing collateral flow and shear stress, particularly in peripheral artery disease (PAD), is supervised exercise (SE). The CLEVER study (**CL**audication: **E**xercise **V**s. **E**ndoluminal **R**evascularization) has proven SE to be even superior to stent revascularization (ST) and sole optimal medical care (OMC) in patients with aortoiliac peripheral artery disease by a significant increase in peak walking distance [46]. Both SE and OMC presented a greater enhancement in high-density lipoprotein (HDL) cholesterol compared with the ST group, due to physical exercise and lipid lowering drugs, respectively. However, looking at the ankle-brachial index (ABI) as a secondary endpoint, only patients with ST showed significantly better results. SE is a non-invasive way of improving PAD. Of note, however, published data on the beneficial effects of supervised exercise lack proof of increased collateral formation as the main underlying mechanism as there is currently no validated method available to quantify collateral formation in the peripheral circulation. 

**Fig. (2):**
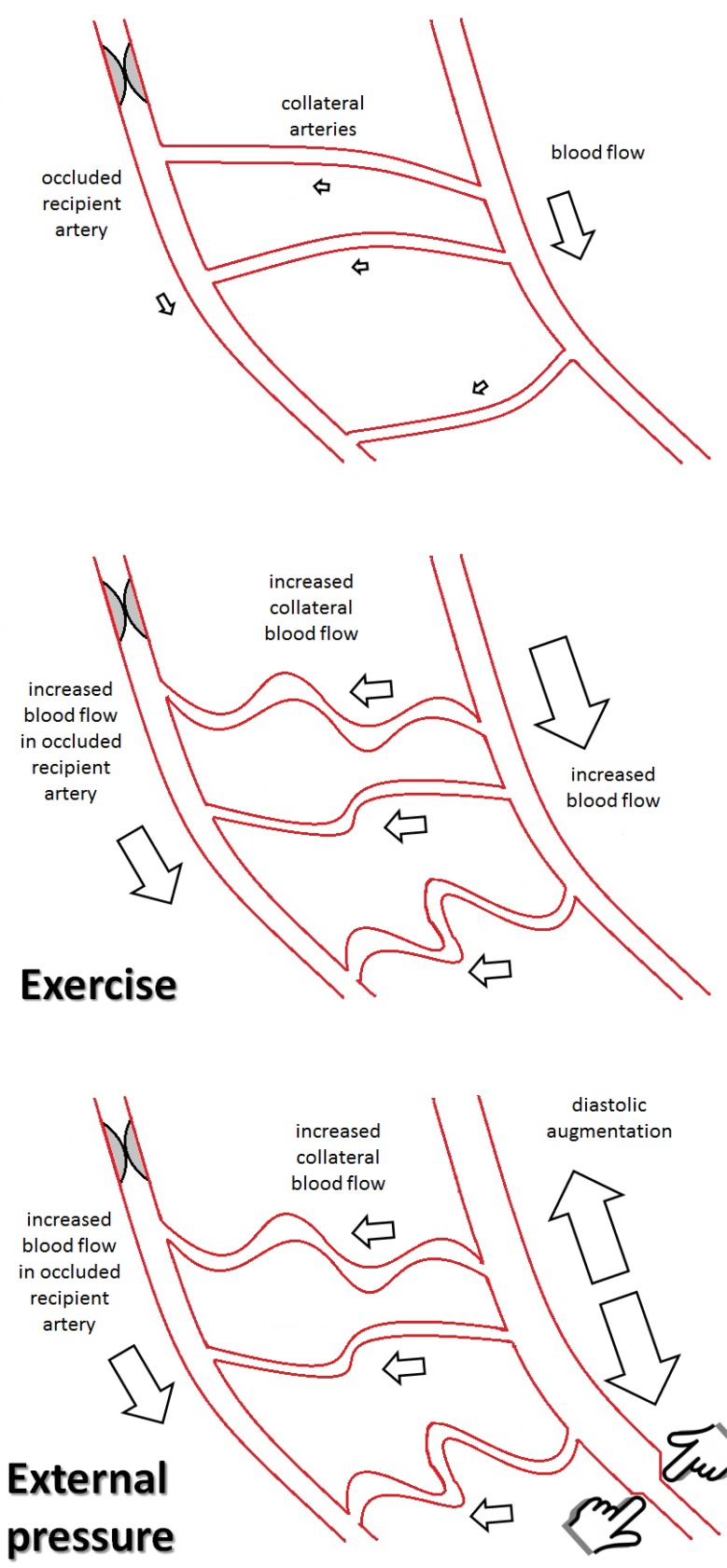
Poor blood flow over small preexisting collateral arteries. The area distal of the occluded artery is not supplied sufficiently. Exercise leads to an increased blood flow in the native artery, causing increased flow in the collateral arteries and arteriogenesis. Blood flow in the occluded recipient artery is improved. External pressure as in ECP leads to increased collateral flow and arteriogenesis through diastolic augmentation, leading to an increase in blood flow in the recipient artery.

Because of similar mechanisms a beneficial effect of exercise can be expected on the coronary circulation as well. In an individual athlete’s report, different levels of physical activity resulted in an increase on both CFI and coronary flow reserve (CFR). At baseline fitness level the healthy sportsman experienced anginal chest pain during a one minute left anterior descending coronary artery (LAD) occlusion, which he did not suffer from at a higher fitness level, suggesting a shear-stress induced arteriogenesis of pre-existing coronary collaterals [47]. A clinical study examining different training modalities and their effects on coronary arteriogenesis is currently enrolling a patient collective [48]. The EXCITE-trial (Impact of Intensive Exercise Training on Coronary Collateral Circulation in Patients With Stable CAD) will be evaluating high-intensity training and moderate-intensity training with optimal medical therapy (OMT) versus OMT only in patients with significant coronary stenosis. Primary endpoint will be the change in collateral blood flow as assessed by coronary catheterization during balloon occlusion. See also (Fig. **2**). 

## ALTERNATIVE IDEAS FROM EXPERIMENTAL MODELS 

Pipp *et al*. for the first time increased fluid shear stress (FSS) by creation of arteriovenous (AV) side-to-side-anastomoses in the porcine peripheral circulation following femoral artery occlusion [49]. Here, AV-shunt results in profoundly decreased arterial pressure (equaling venous pressure) distal to the arterial occlusion, thereby increasing pressure gradient over the pre-existent collateral circulation and consecutively enhanced shear stress. Expression of proarteriogenic genes was markedly increased after the procedure as well as collateral flow. This model which was later repeated in smaller animals [39] for the first time provided evidence, that perfusion restoration following experimental arterial occlusion can exceed 100% of the healthy situation if the stimulus is strong enough. Gene expression analyses identified genes which potentially transduce the mechanical force into collateral artery growth, and application of the corresponding proteins *in-vivo* has been sought to find a means to translate mechanical arteriogenesis into an exogenously applicable pro-arteriogenic drug. Nevertheless, the conception of an arteriovenous shunt is potentially conceivable in the clinical situation as well, where percutaneous or surgical creation of a coronary AV-shunt in a patient with end-stage coronary artery disease and lack of therapeutic options is theoretically feasible. 

As mentioned above, eNOS is induced during arteriogenesis. Past studies have shown that the presence of NO is critical in arterial growth. Application of NO donating moieties has been demonstrated to stimulate arteriogenesis [37]. Substances used in vascular prevention such as aspirin or statins have already been linked to an NO-donating moiety [50] making this approach feasible also in the clinical situation.

Animal studies showed increased flow restoration in a femoral artery ligation model following heart rate reduction by If-channel blockade through administration of Ivabradine. In this study, the augmented pulse amplitude is most likely the key to increased circumferential wall stress and increased arteriogenesis [51]. However, coronary circulation and peripheral circulation differ strongly. For instance, increased pulse wave amplitude has a much higher impact on peripheral vessels than on coronary vessels. In the clinical setting, however, prolongation of diastole as a major hemodynamic effect of bradycardia, results in increased coronary flow and shear stress and can thus likely have a positive effect on arteriogenesis. In fact, low heart rate has earlier been positively related to collateral flow index [52]. A clinical study assessing the effect of Ivabradine on coronary collateral circulation is currently carried out; results are being expected in the course of 2013 (clinicaltrials.gov identifier NCT01039389).

Ivabradine is the first substance assessed for the stimulation of arteriogenesis which has demonstrated proarteriogenic capabilities while attenuating systemic inflammation [51]. Strong inflammatory cytokine prodution has been associated with decreased arteriogenesis, suggesting pro- and antiarteriogenic properties of different inflammatory pathways [53, 54]. Therefore, modulation of these pathways inducing a proarteriogenic milieu rather than induction of full-scale inflammation could be a possible approach for future investigation. 

Cell therapy has gained vast interest in regenerative medicine in the past years. A prospective study on cell therapy for the stimulation of collateral artery growth using quantitative outcome measures (CFI) is currently lacking. One promising approach could lie in the application of vascular progenitor cells. A recent paper has shown the possibility of applying reprogrammed vascular progenitor cells to increase collateral artery growth [55, 56]. This method rather than the use of pluripotent stem cells seems promising and shows a significantly reduced risk of unwanted side effects when compared to stem cell therapy. Also, in earlier investigations, a correlation between circulating progenitor cells and coronary collateralization was discovered [57]. Nonetheless, the multitude of effects of stem cells and the use thereof are still not completely clear and require further research in these fields.

## DOWNSIDES – A WELL-DEVELOPED COLLATERAL CIRCULATION AT ALL COST?

There are possible downsides to a well-developed coronary collateral circulation that should be considered carefully. A meta-analysis comprising seven studies on restenosis after percutaneous coronary intervention (PCI) and drug-eluting stent (DES) implantation shows that good collateralization before intervention is associated with a 40% increase in restenosis when compared to patients with poor collateralization [58]. In another study, a higher recruitable CFI was associated with a better developed collateral circulation and an increase in angiographic restenosis >50% after 9 months [59]. The effects reported could possibly result from reduced antegrade blood flow through the native vessel. A similar effect has been described in collateral arteries after bypass grafting [60]. In a single study, the negative effects on restenosis of a well-developed collateral circulation in patients having undergone bare metal stent (BMS) implantation did not occur [61]. Of note, a good collateralization is associated with increased survival regardless of the increase in restenoses after PCI [62].

## Figures and Tables

**Table 1.** Overview of Studies on Stimulation of Coronary Arteriogenesis. First Column Names the Study, Second and Third Columns Describe the Stimulus Used and Fourth Column Presents the Outcome.

Trial	Stimulus	Substance/route of Administration	Outcome
Seiler *et al*.	chemical	GM-CSF (i.c., s.c.)	Increase in coronary collateralization, increase of CFI
Belardinalli *et al*.	chemical	Dipyridamole (p.o.)	Increase in coronary collateralization, combined with exercise
VIVA	chemical	rhVEGF (i.c.)	No difference in angina frequency or exercise tolerance
AGENT	chemical	Adenoviral FGF4 (i.c.)	Non-significant increase in exercise-time after 4 weeks
FIRST	chemical	FGF2 (i.c.)	No difference in exercise tolerance when compared to placebo
Zbinden *et al*.	chemical	GM-CSF (s.c.)	Increase in CFI, terminated when 2 patients developed ACS
Meier *et al*.	chemical	G-CSF (s.c.)	Increase in CFI, more often lack of ST-segment elevation
Gloekler *et al*.	mechanical	ECP	Increased CFI compared to Sham treatment (augmentation of venous return alone)
Art.Net.-2	mechanical	ECP	Increased CFI and FFR, improvement in angina frequency and dyspnea

## SUMMARY AND CONCLUSION

Because of the impact of ischemic heart disease, a non-invasive approach to stimulate natural bypasses has been subject of extensive research. At the current stage, the focus of coronary arteriogenesis has shifted from cytokine-based therapy towards a mechanical approach where physical exercise and external counterpulsation therapy appear to be most promising means for the stimulation of collateral artery growth. Interestingly, these therapies decrease rather than increase systemic inflammation, questioning the earlier accepted belief that a pro-arteriogenic therapy is always pro-inflammatory. Currently, the results of different studies examining several of the aforementioned approaches are still pending or awaiting clinical trial stage.
